# Long-term population projections: Scenarios of low or rebounding fertility

**DOI:** 10.1371/journal.pone.0298190

**Published:** 2024-04-04

**Authors:** Dean Spears, Sangita Vyas, Gage Weston, Michael Geruso

**Affiliations:** 1 Economics Department, University of Texas at Austin, Austin, TX, United States of America; 2 Population Wellbeing Initiative, University of Texas at Austin, Austin, TX, United States of America; 3 Hunter College and CUNY Institute for Demographic Research, both at City University of New York, New York, NY, United States of America; Jatiya Kabi Kazi Nazrul Islam University, BANGLADESH

## Abstract

The size of the human population is projected to peak in the 21st century. But quantitative projections past 2100 are rare, and none quantify the possibility of a rebound from low fertility to replacement-level fertility. Moreover, the most recent long-term deterministic projections were published a decade ago; since then there has been further global fertility decline. Here we provide updated long-term cohort-component population projections and extend the set of scenarios in the literature to include scenarios in which future fertility (a) stays below replacement or (b) recovers and increases. We also characterize old-age dependency ratios. We show that any stable, long-run size of the world population would persistently depend on when an increase towards replacement fertility begins. Without such an increase, the 400-year span when more than 2 billion people were alive would be a brief spike in history. Indeed, four-fifths of all births—past, present, and future—would have already happened.

## Introduction

All leading global population projections expect the size of the human population to peak in the second half of the 21st century and then decline. But quantitative projections past 2100 are rare, and none quantify the possibility of a rebound from low fertility to replacement-level fertility. Over a decade ago, Basten et al. (2013) [1] presented deterministic “Very long range global population scenarios.” In the years since, global fertility has declined faster than demographers expected. This new information has revised demographers’ expectations. For example, in 2014, Gerland et al. (2014) [2] reported that it was unlikely that the size of the world population would peak within the 21st century, but the most recent United Nations’ central scenario projects a peak in the 2080s.

Approximately two-thirds of the global population now live in a country with a fertility rate that is below replacement level [3]. Declines in fertility to sub-replacement levels can be at least partially explained by improvements in the alternatives to having a child. In many countries, women’s pursuit of higher education and participation in the labor force led to delays in marriage and childbearing that contributed to fertility decline [4, 5]. However, in some countries, like India, fertility has declined to sub-replacement levels despite women having children at young ages [6] and low female labor force participation [7]. In these countries, parents’ desire to invest more in the human capital of fewer children may partially explain fertility decline [8].

An important open question is how likely it is for low fertility to persist. The forces that have led to sub-replacement fertility are unlikely to go away: the alternatives to having a child will likely only improve in almost all countries. Lutz et al. (2006) [9] have influentially suggested that sustained experience with low fertility could result in a “low fertility trap,” once cultural norms, social structures, economies, and policies adapt to low fertility as normal. Quantitatively, Raftery and Ševčíková (2023) [10], estimate a 90% chance that global fertility is below replacement in 2250 and in 2300. Of course, it is not certain that fertility will continue its global downwards trend, nor that fertility must stay below replacement indefinitely. At a minimum, sustained low fertility is plausible enough that it is important to understand it.

Given the prospect of global sustained sub-replacement fertility, this paper presents a series of deterministic, long-term cohort-component projections of the size and age structure of the population under various hypothetical scenarios for future population decline, and potential subsequent rebound. In our first set of scenarios, we consider possible futures in which the world sustains below-replacement-fertility. Then, in our second set of scenarios, we provide what to our knowledge are the first long-term cohort-component projections of global population size that quantify the possibility of a reversal of fertility decline towards replacement-level fertility. All the scenarios that we investigate assume that global fertility stays below replacement for decades at least—consistent with the central projections of nearly all population scientists.

There is substantial uncertainty around how humanity would respond if depopulation persisted for decades. The scenarios we study are not predictions for the future. Rather, they illustrate how the global population could become small quickly, and remain that way despite future rebounds in fertility.

We find that the global population could shrink fast for a range of plausible levels of below-replacement fertility that are already common today. If global fertility rates continue to decline and remain below replacement, we would be living in a brief, unusual spike in human history. A world of many billions of people is an historical anomaly, relative to humanity’s past. It may also be an anomaly, if fertility rates stay low, relative to humanity’s future. We then quantify the consequences for long-run population dynamics of various possible future transitions to replacement fertility. By construction, these scenarios result in stabilization of the period population size. But that size depends radically on when the transition begins, which determines how much depopulation precedes it. For example, if the transition towards replacement fertility begins in 2125, the stationary population size would be about 8 billion, but if the transition begins just fifty years later in 2175, the stationary population size could be between 2 and 4 billion, depending on how low fertility initially falls. And if no transition to replacement fertility happens within the next few centuries, the size of the global population would become very small.

Our findings update and extend the literature on long-run population projections. In 2004, the United Nations Population Division published long-range population projections to 2300 that projected long-run stabilization of the global population at about 9 billion [11]. Nine years later, Basten et al. (2013) [1] called into question the assumption of long-run population stabilization, recognizing that fertility rates in Europe were below replacement and had been there for several decades. Since then, global fertility has declined faster than demographers predicted and many more people live in countries with sub-replacement level fertility. The UN, however, has not updated its long-run projections to reflect the most recent two decades of data. This paper builds on the work of Basten et al. (2013) [1] by incorporating and extending population science’s updated expectations for future fertility rates. We also quantitatively explore the consequences of any hypothetical future *reversal* towards replacement fertility, which no other long-term population projections explore.

## Materials and methods

We construct deterministic, cohort-component method population projections for each of the “countries or areas” of the 2022 United Nations World Population Prospects (WPP) and aggregate these to worldwide totals. We use the demographic rates of the WPP Medium projection that omits migration, until it ends in 2100. Beyond 2100, we make assumptions for long-run fertility and mortality.

The core independent variable of our study is a *long-run total fertility rate* towards which the entire world converges after the end of the WPP projection in 2100. In our first set of results, we use a range of long-run future global fertility rates that are below replacement level. Our deterministic projections are intended to produce *conditional* results, answering the hypothetical question “What would happen to the global population *if* fertility in the future takes these possible paths?” Although it is not certain that long-run fertility will remain below replacement level in the long run, it is plausible enough that it is worth understanding.

The long-run fertility scenarios that we study are based on several broad patterns of the past decades and centuries. These include: the fact that the global average birth rate has fallen significantly over the past two centuries; the fact that countries that have fallen below replacement fertility have in almost all cases remained below replacement; the fact that in regions where fertility is currently high it is currently falling; and the fact that more than two-thirds of people in the world now live in a country with below-replacement fertility. These broad patterns motivate the conditional projections we present. We do not assign confidence intervals to these outcomes or derive a single projection from a more complicated mathematical extrapolation, because to do so would communicate false precision about the probability of what is a fundamentally conditional exercise.

In each five-year time step, each country’s age-specific fertility rates either increase or decrease by 3% until the country’s total fertility rate (TFR) reaches the specified long-run TFR. Because all countries approach the long-run TFR, the world does, too. In the limit, each country retains a slightly different age pattern of fertility because each has a different age pattern of fertility in 2100 at the end of the UN projection. Although fertility decline in many countries has been accompanied by a delay in childbearing, there is precedence for countries reaching low fertility yet maintaining different age-patterns of fertility. For example, India’s TFR is now 2.0 births per woman, yet women continue to begin childbearing at young ages [6], a pattern which is in stark contrast to what has occurred in high-income countries. S2 Fig shows scenarios for age-specific fertility rates globally and for selected countries.

The result of this procedure is that in the limit each country has the same average completed cohort fertility. Our 3% assumption for the pace of fertility convergence is conservatively slow compared to the change in global TFR, which decreased by roughly 7% over the five years from 2017–2022. However, the actual future rate of change could be faster or slower, so we show in S1 Fig that our descriptive results are robust to other plausible speeds. The Supporting Information describes the fertility assumptions in further detail.

Population projections also require assumptions for future mortality rates. After the WPP projections end in 2100, we assume that mortality rates everywhere converge to a life expectancy at birth of 100 years. We implement this high life expectancy as a demonstration that our depopulation main results are robust to mortality reductions. There is considerable uncertainty regarding future declines in mortality, however, and results shown in Supporting Information (S5 and S6 Figs) demonstrate that our results are robust to alternative assumptions for mortality. S4 Fig shows the assumed age-pattern of mortality in various periods until life expectancy at birth of 100 years is reached globally, and the Supporting Information describes the mortality assumptions in further detail.

**Fig 1:**
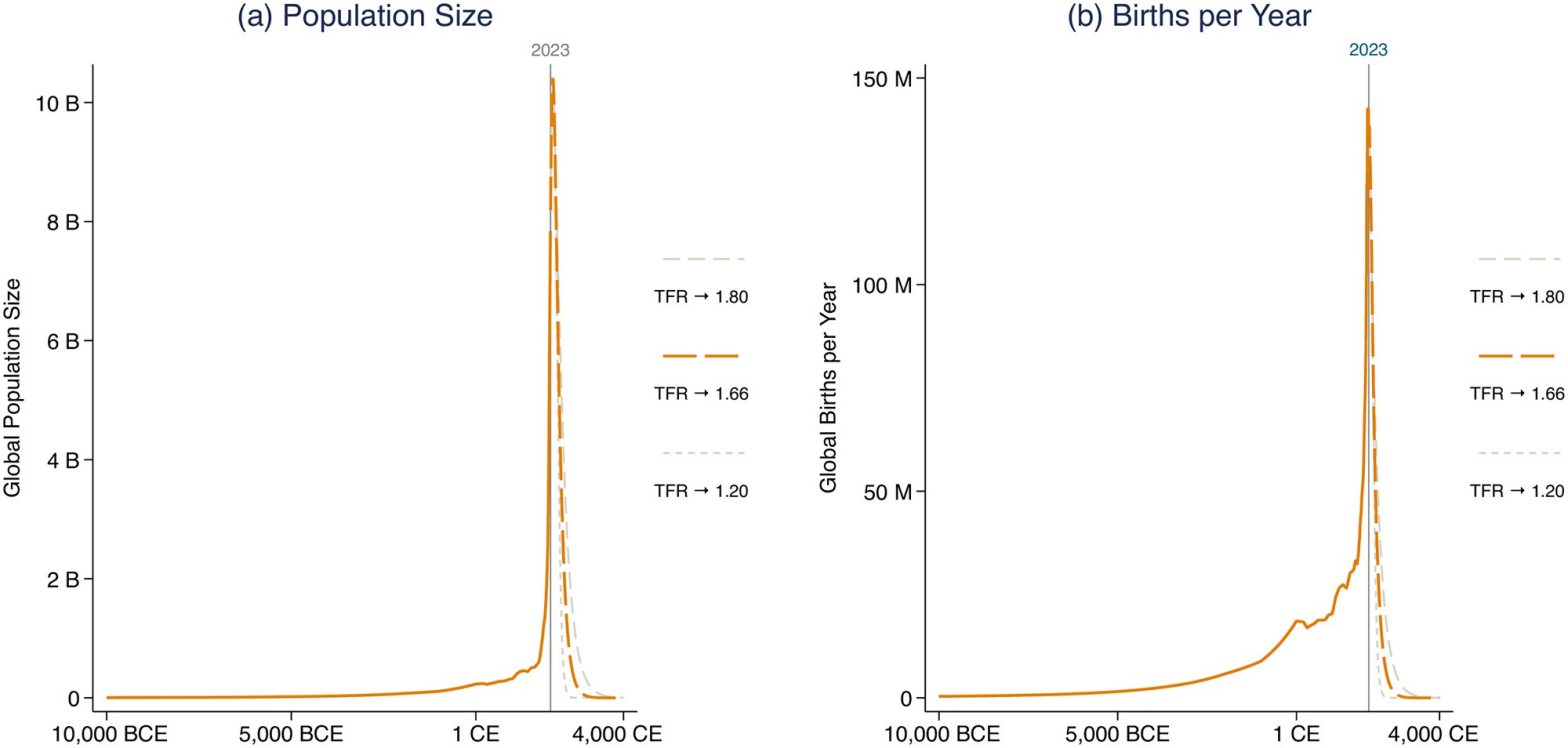
Global population as a spike in world history. Historical data are taken from Kaneda and Haub (2022) [12]. Projections from the present to 2100 are the Medium projection of the 2022 UN World Population Prospects. Projections from 2100 are produced by the authors as described in the Methods. “TFR → 1.66,” “TFR → 1.8,” and “TFR → 1.2” indicate three hypothetical scenarios for long-run global fertility rates towards which the world converges.

Our cohort-component projections provide age-specific quantification for each five-year time step of births, deaths, and the age pyramid of the population. All results below reflect this methodology, and it is the basis of the Spike diagram in Fig 1. Because our dependent variable of interest is the size and age structure of the *global* population, we ignore migration. This focuses on our independent variable of interest: possible futures for global fertility. In this we follow Basten et al. (2013) [1] who “assume a slow phasing out of international migration,” summarizing that “this is clearly unrealistic,… but has little effect on global population size, which is the main point of interest here.” Indeed, the difference in global population size between the WPP Medium no-migration variant and the WPP Medium variant with migration is only 12 million people by 2100, or about 0.12%. This is a very small difference.

Our second set of results describes possible hypothetical scenarios for future fertility increases up to the replacement level. A fertility increase scenario has two phases: first, the initial convergence after 2100 towards a long-run fertility rate (as described above); and second, a transition in a specified year towards replacement fertility. In the long-run the second phase approaches a stationary population with fixed fertility and mortality rates and zero growth. Therefore, a fertility increase scenario is described by two independent variables: the initial long-run TFR (such as 1.5) and the year in which the long-run TFR changes to replacement (such as 2150). This allows us to quantify long-run consequences—that is, path dependency—of the date at which fertility reverses and begins increasing. How, we ask, does the long-run size and structure of the population depend upon the year in which fertility rates reverse towards replacement levels? Methods are described in further detail in the Supporting Information.

## Results

Fig 1 presents our main results. The figure plots the size of the global population a long way into the past (from 10,000 BCE) and a shorter way into the future. Historical data are taken from Kaneda and Haub (2022) [12]. Projections from the present to 2100 are the Medium projection of the 2022 UN World Population Prospects. Projections beyond 2100 make various assumptions on global average fertility, as indicated. The figure shows that, according to our projections (which follow the UN central scenario for the years of overlap), we would be living in a brief, unusual spike in human history. Population sizes that are familiar today are an exception, relative to both the past *and* the future.

To make these ideas concrete, consider the post-2100 projection in Fig 1 that assumes global fertility converges to a total fertility rate (TFR) of 1.66, which is selected for familiarity because it is the period TFR of the US in 2020. About a third of people alive now live in a country with a lower period TFR than 1.66. So Fig 1 assumes that fertility in those places would increase—despite that the trend in all large regions of the world, including where fertility is already below replacement, is stable or falling fertility. The other dashed lines, tracing scenarios after 2100, alternatively assume that the world converges to the period TFR of present-day East Asia (1.2) or present-day Mexico (1.8). We present these three alternatives not as predictions for future fertility, but to illustrate that, while the differences among these possible fertility rates are important, they are not very important for the shape of the spike in Fig 1. In all cases, we now would be living through a brief and sharp peak in population size.

Although population momentum bounds the population sizes that would be feasible in the coming few decades, global depopulation in the 22nd century and beyond could be rapid—just as rapid as the population explosion of the last 200 years—because exponential growth and decay are governed by the same compounding dynamics. Fig 1 shows scenarios in which only 26 billion human births would remain to happen in the future (interpreting the projection literally). 120 billion births have already occurred. This would imply that four-fifths of all the people who will ever live have already been born. (Such a scenario assumes no fertility rebound at any point, an assumption we alter for the second set of scenarios we evaluate).

**Fig 2:**
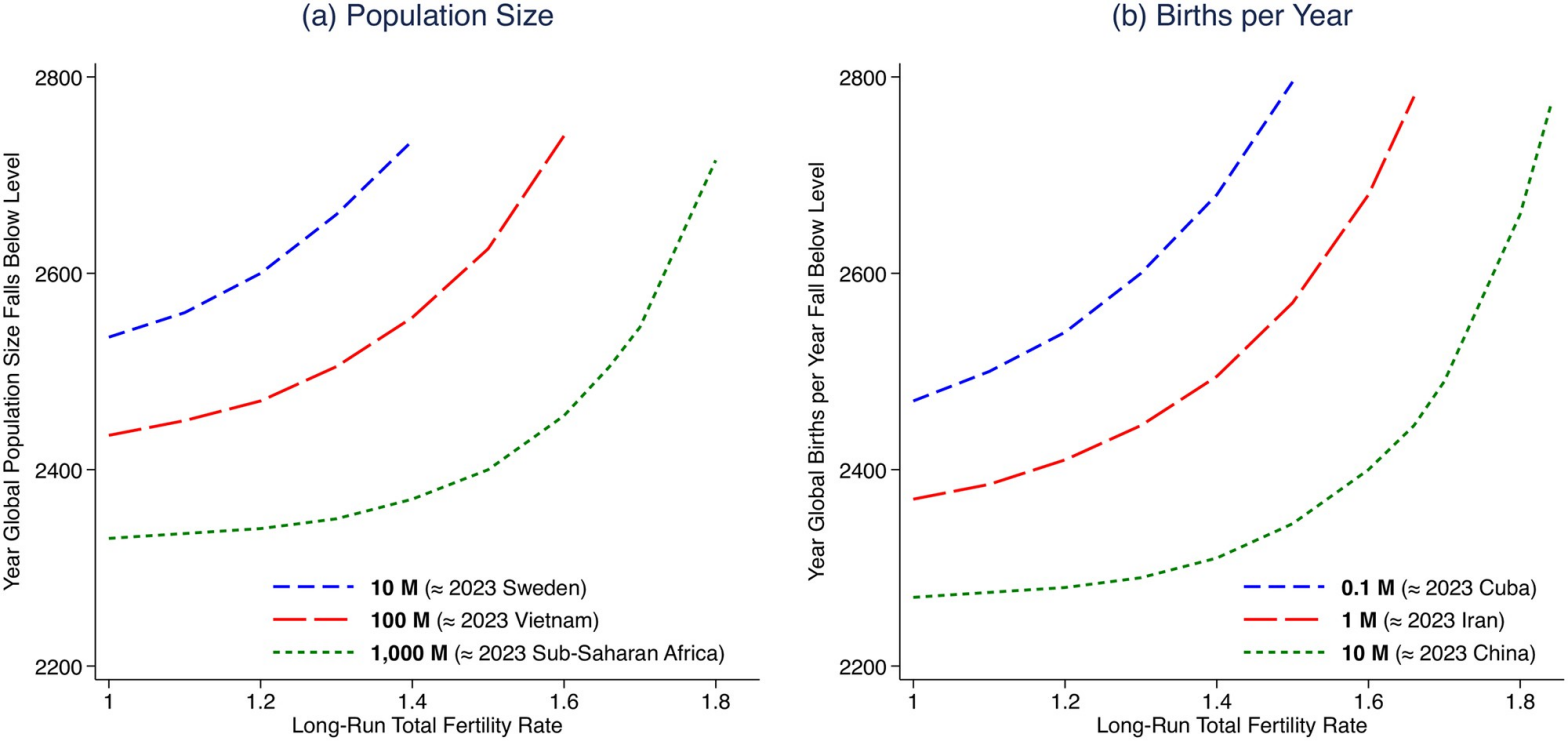
Longest-term consequences for population size of alternative long-run global fertility rates. (a) plots iso-population curves and (b) plots iso-births curves. Each line indicates the combination of global fertility rates and dates that result in the indicated population or annual birth milestone. For example, if global fertility converges to 1.4 births per woman, then there are projected to be 1 billion people in 2370 and 1 million births per year in 2495.

Fig 2 is an expansion on the same family of population projections shown in Fig 1. Whereas Fig 1 considered hypothetical scenarios for total fertility converging to 1.20, 1.66, and 1.80, Fig 2 displays the implications of convergence to any long-run TFR between 1.00 and 1.80, as indicated along the horizontal axis.

Panel (a) of Fig 2 traces a set of iso-population lines, indicating which combinations of global fertility rates and dates would yield the indicated population milestones. It demonstrates the delay in falling below a particular population milestone associated with increases in long-run TFR. A steeper curve indicates a longer delay, and a flatter curve indicates a shorter delay.

For example, what would happen if the world converges to a TFR of 1.2, the average fertility rate that East Asia, taken as a whole, exhibits today? A vertical line traced up from TFR = 1.2 crosses the (short dashed, green) iso-population curve for 1 billion people in 2340. Thus, within 250 years of the lifetime of most children born today, there would be fewer than 1 billion people alive. Panel (b) tells us that by 2280, there would be only about 10 million babies born each year, compared to about 135 million births in 2022. Only four decades after the global population reached 1 billion, by 2380, it would further halve to 500 million; this halving time of 40 years would be comparable to the world population’s doubling time of 37 years in the mid-20th century. If, instead, global fertility converges to 1.5 babies per woman (like in today’s Europe), these milestones would be delayed by only 25 and 95 years, for the 1 billion and 500 million thresholds, respectively.

Figs 1 and 2 present population projections under the assumption that fertility rates for each country change by 3% each five-year period, and that mortality rates decline such that long-run life expectancy at birth is 100 years. S1 Fig shows that the main findings on the speed of depopulation are qualitatively similar under different assumptions for the pace of change in fertility rates. Similarly, S5 Fig shows that the depopulation results are very similar even if we assume an even higher long-run life expectancy at birth of 120 years.

**Fig 3:**
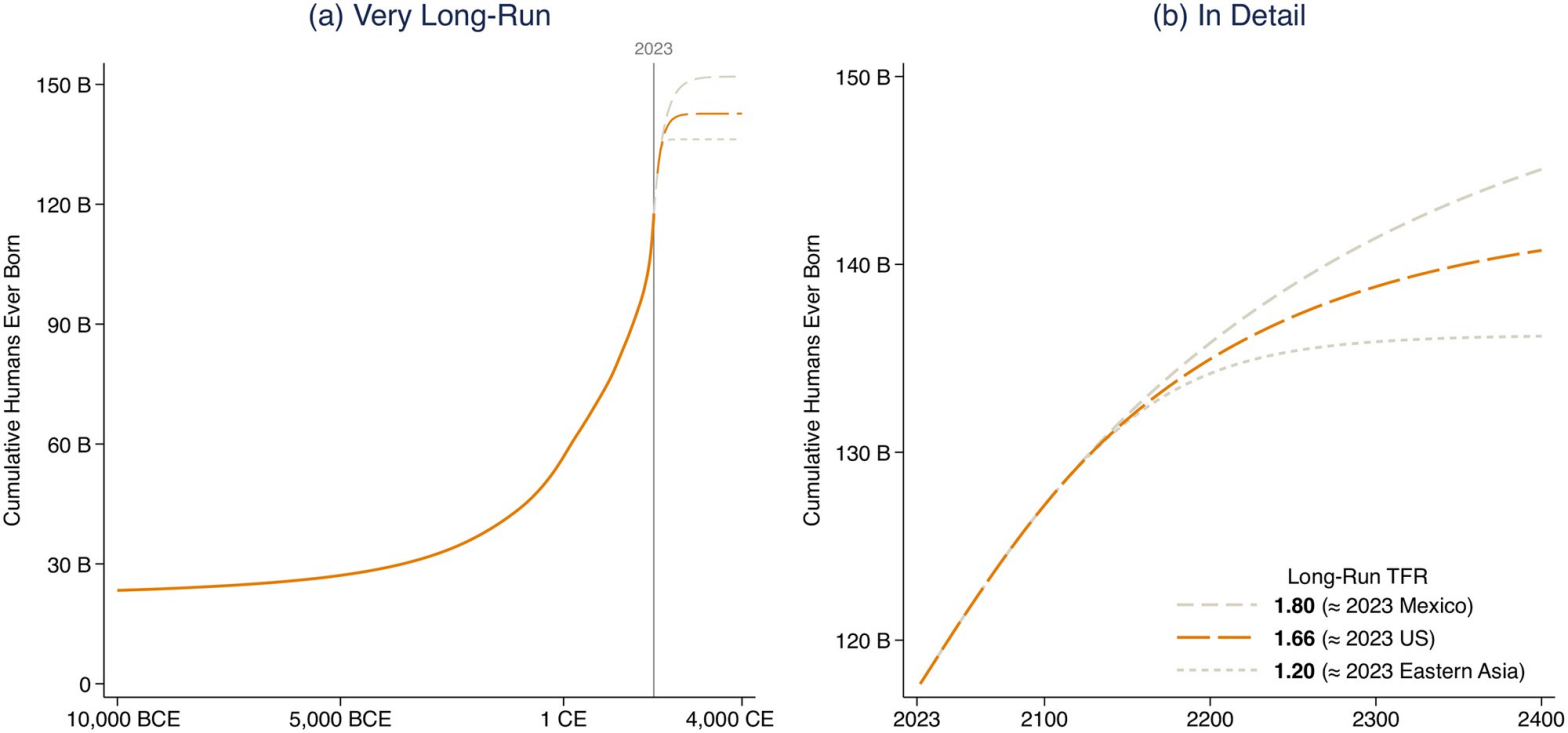
Cumulative count of humans ever born, as a function of long-run global fertility. Fig 3 uses the same set of population projections as Figs 1 and 2, but tabulates the projection as a cumulative count of human births. If fertility rates stay below replacement, then the total number of human births is projected to stay below 150 billion, or 26 billion more than have so-far occurred. (a) shows cumulative births in the very long-run, and (b) zooms in on cumulative births in the next couple of centuries.

Fig 3 documents the consequences for the total number of humans who ever live; it is equivalent to the integral under panel (b) of Fig 1. So far, there have been about 120 billion people born [12]. If fertility stays below replacement—if global TFR indeed asymptotes to the levels that we consider—then Fig 3 shows that this count would never exceed about 150 billion, and could be less. In that case, at the time that we are writing, 80% of births would be in the past. As panel (b) demonstrates, low fertility would not have to continue for very many centuries (or decades) for humanity’s count of births to approximately reach its cumulative limit.

Of course, the further that future population sizes fall away from the sizes that are familiar to the population science community, the less informative these results can be about what might happen. These are merely deterministic population projections, conditional on our hypothetical scenarios. They are intended only to illustrate the consequences of sustained low fertility rates. Population science’s cohort-component model was not created to specify what exactly would happen when humanity’s numbers become very small, so that situation is outside the scope of our research. What the model can tell us is that if low fertility is sustained for just a few centuries, then the population size could be quite small.

**Fig 4:**
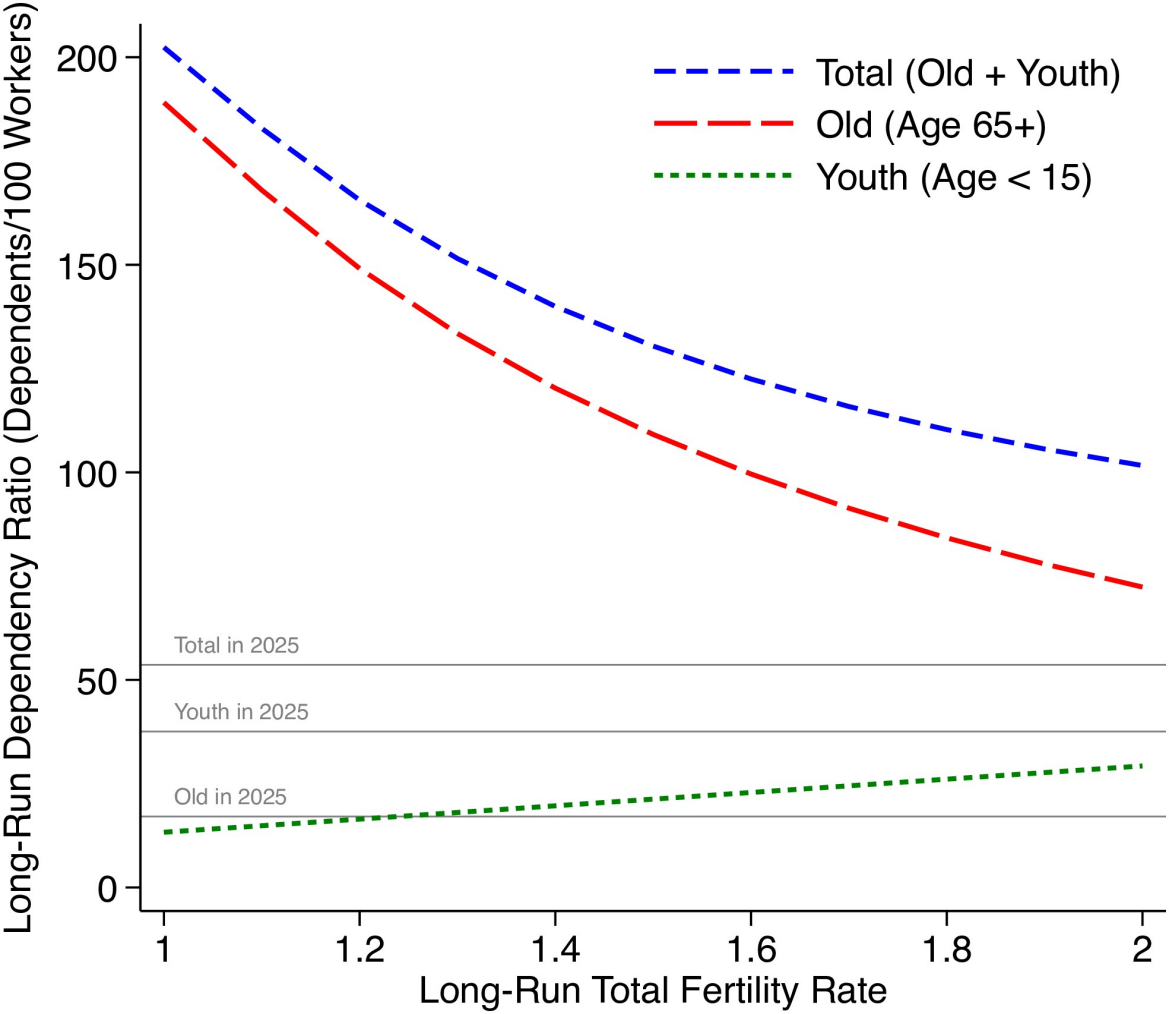
Consequences of long-run fertility for long-run dependency ratios. Fig 4 uses the same set of population projections as Figs 1 and 2, but presents the consequences for dependency ratios as a function of the assumed scenario for long-run, asymptotic fertility.

Fig 4 describes consequences for the age structure of the population that the world would converge to. The youth dependency ratio would fall, because there would be few births. The old-age dependency ratio would become quite large under the fertility rates that are common today in Europe: There would be six or more times the number of older adults per working-age adult as there are now. In 2019, the highest country-level old-age dependency ratio in the world was Japan’s, with about half an older adult per working-age adult; if the world converges to a TFR of 1.2, like East Asia today, then it would converge to an old-age dependency ratio of 150, or three times that in Japan in 2019. Marois et al. (2021) [13], Galor (2022) [14], and others have emphasized that improving human capital and changing labor markets could soften the economic consequences of these changes in the dependency ratio. This is true; and yet, some forms of social interaction or in-person care work may not have good substitutes within a few centuries. It is also possible that in the far future, health improvements might contribute to longer working lives. This would imply lower dependency ratios than displayed in Fig 4. It is beyond the scope of this article, however, to predict how future societies will define the working ages. Fig 4 simply displays future dependency ratios *if* people continued to stop working at age 65, on average.

**Fig 5:**
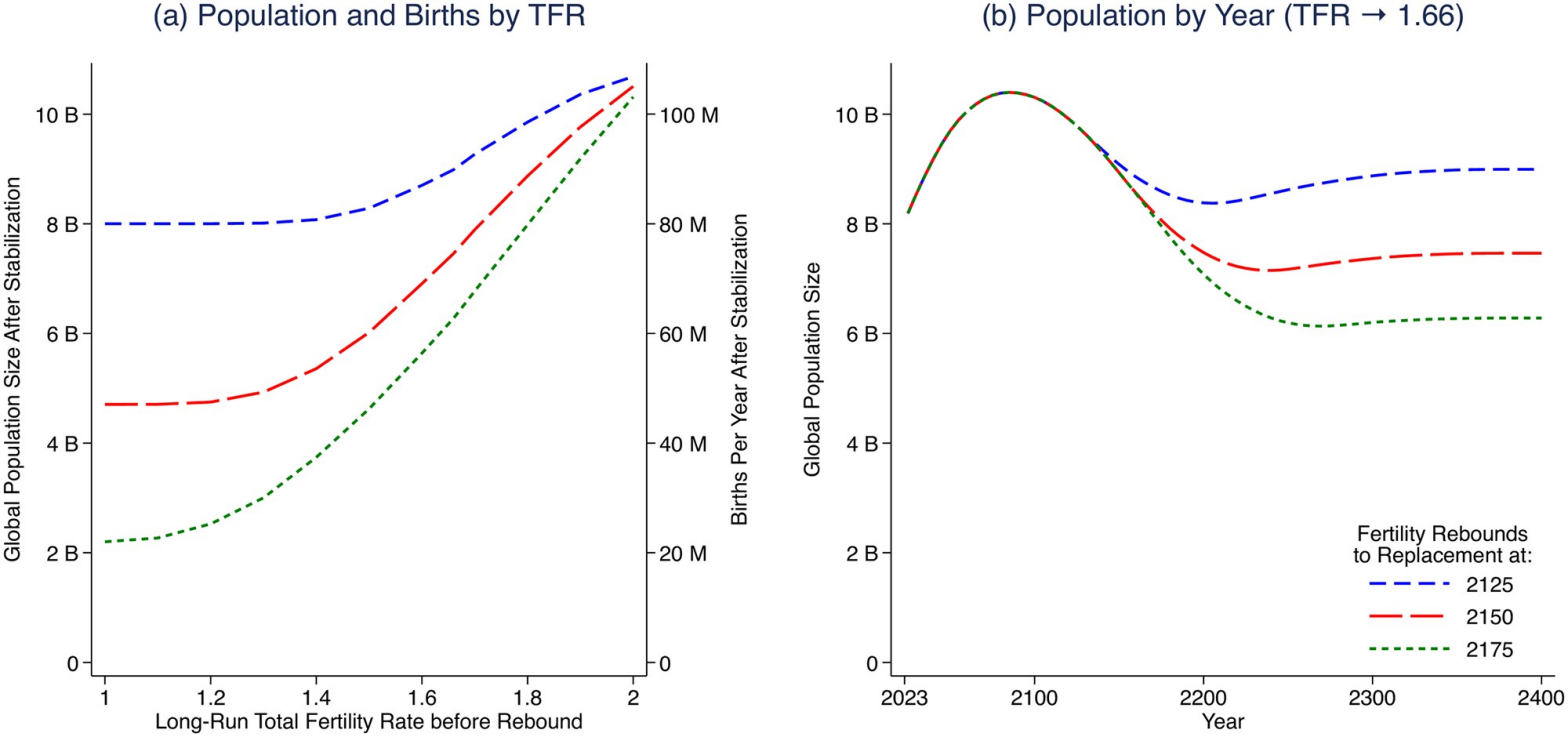
Future stationary population size depends on when fertility increases to replacement. (a) displays global population size (left) and annual births (right) after the population has stabilized. TFR first falls toward the given level after 2100 and then rises to replacement at either 2125, 2150, or 2175. (b) displays population size over time if the long-run TFR before increasing to replacement level is 1.66, demonstrating how even a few decades change in timing of this hypothetical transition could alter long-run population sizes by billions.

Our second set of future fertility scenarios assumes that, at some point in the 22nd century, the global long-run fertility rate changes to replacement fertility. Here the research question is how long-run outcomes depend on *when* this transition occurs. Fig 5 plots three scenarios for when the reversal towards replacement fertility begins, crossed with a range of possible 22nd century global fertility rates (from 1.0 to 2.0) towards which the world converges before fertility begins to increase back to replacement. The left vertical axis of panel (a) indicates the stationary population size, in the long run after the world has converged to replacement rates. The right vertical axis indicates the annual number of births worldwide, again in the long run after the world has converged to replacement rates.

Panel (a) of Fig 5 shows that the timing matters, because decades or centuries spent depopulating would shape the population size that future replacement fertility would eventually stabilize to. Notice that, unlike in our main results of continuing depopulation, here we describe convergence to a stationary population; as a result, the ratio of the population size to the annual number of births in the limit is fixed at the life expectancy at birth, as the two vertical axes of Fig 5 Panel (a) demonstrate.

The results reveal an interaction between the initial, pre-increase long-run target fertility level, on the one hand, and the year that the fertility increase towards replacement begins, on the other. If the increase starts early (in 2125) then not much time is spent at low fertility, so the initial fertility rate does not make as large a difference to the long-run size of the population. But if the increase starts even fifty years later (in 2175), then the long-run size of the population would be as low as 2 to 4 billion, if the initial fertility rate was low, rather than around 8 billion if either the initial fertility level was higher or the increase started earlier. So, even if depopulation is halted by a future increase in global fertility, the time spent depopulating could make a permanent difference in the size of the population, depending on how long fertility was low. A few decades of timing could result in a multi-billion difference in a permanent, stationary population size. S6 Fig shows that this result is robust to alternative mortality assumptions.

Panel (b) of Fig 5 examines our central case of TFR initially converging to 1.66. Here, a one-year delay in the initiation of a fertility rebound would cause an approximately 0.7% permanent reduction in the stationary size of the long-run population. Equivalently, delaying the rebound by one generation (30 years) reduces stabilized population size by about 19%. Despite its significant implications for the long run population size, the timing of a fertility rebound to replacement would have no impact on the long-run age structure or dependency ratios of the population in these scenarios, because these quantities (in the assumed stationary populations) are invariant to population size.

## Discussion

This article contributes long-run population projections under future scenarios in which global fertility converges and remains at below-replacement levels, or increases to replacement levels. If fertility remains below replacement in the long-run, then the population spike of this century will be a brief anomaly relative to humanity’s past and future. If, instead, global fertility rates reverse course to increase towards replacement level at some point in the future, the length of time spent depopulating has important implications for the long-run global population size.

Returning to Fig 1, if these projections are informative, then the year with the greatest count of births would already be in the past. Panel (a) of Fig 1 tallies population size. Its peak occurs slightly to the right of the vertical line indicating 2023: The demographic consensus is that world population will peak between 2060 and 2090 [3, 9, 10, 15]. Panel (b) counts births per year. There, the peak has already occurred. The UN estimates it happened in 2014.

The most important limitation of this study is that its projections are deterministic; they are *conditional* statements that would describe the world *if* it followed a path similar to one of the stylized scenarios. No formal method could accurately predict what would happen once the world population size, in fact, became small. Instead, our study should be read narrowly as a quantification of how quickly the global population size could change under sustained below-replacement fertility, and how much timing matters for any future rebound to replacement.

This limitation suggests a question, beyond the scope of our study: Is sustained below-replacement fertility plausible? The population science literature suggests so. In a survey, many population scientists expected low future fertility [16]. In the Human Fertility Database (Max Planck Institute for Demographic Research (Germany) and Vienna Institute of Demography (Austria)), the completed cohort fertility of cohorts born since 1950 has fallen below 1.9 for 27 countries; so far, in none of these has it ever subsequently risen to 2. Arenberg et al. (2022) [17], pooling data from many recent Demographic and Health Surveys, emphasize that fertility decline is found in essentially every population and sub-population, including, for example, sixteen informative subdivisions of India, a country now emerging as a below-replacement-fertility population. Vogl (2020) [18] shows that the aggregate consequences of intergenerational correlations between parents’ and children’s fertility is quantitatively insufficient to meaningfully increase overall, population-level fertility rates [*cf.* 19]. Gietel-Basten (2019) [20] and others document that pro-natalist policies in low-fertility countries have generally done little to sustainably increase cohort completed fertility (although, of course, population control polices have often done considerable harm while achieving little [21–23]).

Though it is not the aim of this paper to consider the implications of a small and shrinking population for global well-being, other than in terms of implications for the old-age dependency ratio, we note that a substantial literature has linked the population size and growth rate to macroeconomic growth and average living standards—see Jones and Romer (2010) [24] for an overview.

Taken together, this collage of social science evidence suggests the importance of considering scenarios of sustained low fertility. We contribute new quantitative scenarios to better understand this possible future.

### Method of projecting population

We use the cohort-component method (CCM) for projecting age- and sex-specific population size in five-year periods for 236 countries [25]. The CCM takes as inputs period age-specific fertility rates, age- and sex-specific person-years lived, and sex-ratio at birth. Using this data, it estimates every country’s population size for each sex by age-group and period. Our projection method replicates the United Nation’s World Population Prospects (WPP) “zero-migration” variant up to 2100, when the UN projections end. Our method projects global population will be 10.325 billion by 2100 while the WPP zero-migration and medium variants project it will be 10.337 billion and 10.349 billion, respectively. The differences between our projections and the UN’s are small: 0.11% for the zero-migration variant and 0.23% for the medium variant. We suspect that these small differences are driven by rounding and the way we combined annual data into 5-year periods, rather than modeling differences.

#### 2025 to 2100 fertility, mortality, and sex-ratio at birth

We calculate population from 2025 to 2100 using projections from the WPP 2022 “zero-migration” variant [3]. Although unrealistic, our assumption of no migration, following Basten et al. (2013) [1], does not affect our conclusions about global population size and age-structure. Indeed, the difference in global population size between the WPP zero-migration variant and the WPP medium variant with migration is only 12 million people by 2100, or about 0.12%. After 2100, we generate our own trajectories for fertility, mortality, and sex-ratio at birth, using methods which we describe in greater detail below.

WPP projections are available at https://population.un.org/wpp/Download/Standard/CSV/. Here, we list the WPP variables that we use and the files that we obtained them from:

1. Population: Population on 01 July, by five-year age groups (2022–2100, other scenarios)
2. Age-specific Fertility Rate (ASFR): Fertility (1950–2100, five-year age groups)
3. Person-years lived (_5_L_x_): Abridged life table (2022–2100, medium)
4. Sex-Ratio at Birth (SRB): Demographic Indicators (2022–2100, other scenarios)

The WPP provides data on an annual basis. To increase the speed of our program, we combine individual years into five-year periods. Population at the beginning of the period remains the same while fertility, sex-ratio at birth, and person-years lived are the average of the starting year of each period and the start of the following five-year period.

#### Fertility rates after 2100

Our main results present hypothetical global population sizes in the long-term future under different assumptions for long-run fertility rates. Beginning in 2100, total fertility rates (TFR) in each country converge to a long-run TFR. This is achieved by increasing or decreasing age-specific fertility rates (ASFR) by a given percentage in each five-year period. There are therefore two variables that determine the trajectory of fertility rates: the long-run TFR, and the speed of convergence to the long-run TFR.

S1 Fig provides examples of global fertility rates and corresponding population sizes across periods under various long-run TFR targets and convergence speeds. “Very Slow”, “Medium”, and “Very Fast” convergence speeds correspond to a 1%, 3%, and 5% rate of absolute change in TFR per five-year period. Comparing population size across different convergence speeds shows that population size is not highly sensitive to the speed of TFR convergence. The main results in the paper use a “Medium” convergence speed, which is roughly the rate at which South-eastern Asia’s TFR changed between 2017 and 2022.

In the long run, ASFRs in the model differ across countries, despite that all countries reach the same TFR. S2 Fig displays projections of age-specific fertility rates in India, Nigeria, the Republic of Korea, and globally, from 2025 until 2150 under the 1.66 births per woman scenario. Differences in long-run ASFRs arise in the model because each country is projected to have a distinct age-profile of fertility in 2100. We believe that this is a feature rather than a bug of the model. Although fertility decline in high-income countries has been accompanied by a delay in childbearing, not all countries that have below-replacement fertility have undergone the same transition in the age-profile of fertility. For example, India’s TFR is now 2.0 births per woman, yet women continue to begin childbearing at young ages [6], a pattern which is in stark contrast to what has occurred in high-income countries.

#### Rebound fertility scenarios

We also present results on stabilized population size under hypothetical scenarios in which future fertility rebounds to replacement level. In these scenarios, we first assume that fertility rates approach a given long-run fertility rate after 2100, as described above. Then, in a specified year, we assume that fertility rates begin to “rebound” to replacement levels. There are two parameters that describe these scenarios: the long-run global fertility rate after 2100 before rebounding to replacement level, and the year in which the rebound to replacement level begins. These scenarios use a long-run life expectancy of 100 years and a “Medium” TFR convergence speed of 3% per five-year period.

Fig 5 panel (b) in the paper vary the year in which the rebound to replacement level begins and hold constant the long-run global TFR after 2100. The results show that the long-run stabilized population size is very sensitive to the year in which the rebound begins. Here, we vary the long-run global TFR after 2100, and hold constant the year in which the rebound to replacement begins. S3 Fig shows TFR and world population given various baseline long-run TFRs followed by a rebound to replacement starting in 2200. This figure demonstrates the sensitivity of the resulting stabilized population size to the fertility rate preceding a transition to replacement. Therefore, the long-run stabilized population size is highly dependent on *both* the TFR prior to the rebound, and when the rebound begins.

#### Mortality after 2100

Our main results assume that mortality rates continue to decline after 2100, and that life expectancy at birth (e_0_) for males and females converges to 100 years in all countries. After 2100, life expectancy for each sex in each country increases by the same increment as it did between 2095 and 2100, the final five-year period of the WPP projections. Since in most countries, life expectancy at birth for females is higher than for males, females reach the maximum possible life expectancy sooner than males. Life expectancy at birth (e_0,i,s,t_) in country *i* for sex *s* in time-period *t* in periods after 2100 can be described by the equation:

$$\begin{array}{c} e_{0,i,s,t}=e_{0,i,s,t-5}+\Delta e_{0,i,s,2100} \end{array}$$ (1)

where Δ*e*_0,*i*, *s*, *t*_ is the change in e_0_ between period *t-5* and *t*.

We follow two steps to allocate this overall mortality decline across age groups. Because the cohort component method uses person-years lived in each age range (_5_L_x_) as an input, we modify this demographic quantity directly. _5_L_x_ for each age, sex, and country, in each time period *t* is calculated as:

5Lx,t=5λx,t+5δx,t (2)

where _5_λ_*x,t*_ is defined as

5λx,t=5Lx,t-5+Δ5Lx,t-5 (3)

and *δ* is an additional term that is described below. Δ_5_*L*_*x*,*t*−5_ is the change in _5_L_x_ for that age range between period *t-10* and period *t-5*.

_5_λ_*x,t*_ is subject to the following constraints:

1. _5_λ_0,*t*_ cannot exceed five, the number of years in the period.
2. _5_λ_*x,t*_ for age-groups between 5–99 cannot exceed _5_*L*_*x−*5,*t*_, the number of person-years lived in the next youngest age group.
3. _∞_λ_100,*t*_ can only exceed _5_*L*_95*t*_ if the maximum life expectancy at birth is 105 years or greater. For example, if the life expectancy maximum is 90 years, then _∞_λ_100,*t*_ cannot exceed _5_λ_95*t*_. Conversely, if the maximum is 110 years, then _∞_λ_100,*t*_ in the oldest age group cannot exceed 10.

When _5_λ_*x,t*_ for a particular age group is constrained by the criteria listed above, we redistribute the amount above the designated maximums described above from the above-maximum age group to below-maximum age groups. This enforces the age-specific maxima described above while still maintaining the same incremental increase in life expectancy at birth each period as described in Eq 1. The reallocation process is such that age groups that are furthest from their maximum receive a larger proportion of person-years. This achieves the pattern of slowing mortality reductions as mortality declines in an age group.

We define _5_*maxL*_*x,t*_ as the maximum possible _5_*L*_*x,t*_ for the age group *x* to *x+5* in period *t*, based on the constraints listed above. We also define *α* and *β* based on the following formulas:

5αx,t=max{0,5λx,t-5maxLx,t} (4)

5βx,t=max{0,5maxLx,t-5λx,t} (5)

such that *α* is greater than zero only when the allocation for the age group is above the maximum possible allocation for that age group, and *β* is greater than zero only when the the maximum possible allocation for that age group is above the allocation for the age group. Then _5_*δ*_*x,t*_ is defined as follows:

5δx,t=∑a=0∞5αa,t×5βx,t∑a=0∞5βa,t (6)

S4 Fig panel (a) displays the result of the processes described above. It displays _5_L_x_ in each age group by age for various periods between 2025 and 2400, under the mortality scenario in which life expectancy at birth increases to 100 years in all countries.

The main results assume that life expectancy at birth continues to increase until 100 years. We also explore the robustness of our results to an alternative mortality assumption in which life expectancy at birth increases to 120 years. S4 Fig panel (b) displays the two scenarios for life expectancy at birth.

S5 Fig compares global population size under various long-run TFR and life expectancy scenarios. The figure makes clear that population size is much more sensitive to differences in TFR compared to (even large) differences in life expectancy at birth. Part of the reason for this is because after 2100, much of the reduction in mortality occurs in older ages, after the reproductive years. This implies that the main results on depopulation are robust to different mortality assumptions.

S6 Fig shows the robustness of the rebound exercise to different assumptions for life expectancy at birth. The y-axes show the stabilized global population size under the assumption that life expectancy at birth reaches 100 years (the axis on the left), and 120 years (the axis on the right). The three different lines in the figure represent the same scenarios as in the main text for when the rebound to replacement fertility occurs. The notable feature in this figure is that the left-hand and right-hand axes are very similar, meaning that the different assumptions for life expectancy do not substantively change the main take-away of the rebound exercise.

#### Sex ratio at birth after 2100

Beginning in 2100, each country’s sex-ratio at birth (SRB) increases or decreases by 0.5% per five-year period until reaching the WPP’s projected 2100 global average SRB of 1.045 males born per female birth. Most countries reach this SRB by the mid-22nd century. The convergence in SRB ensures that our long-run fertility assumptions produce similar population growth rates across countries. As most countries’ SRB are within a few percent of each other to begin with, our assumption for SRB does not qualitatively alter the conclusions of the paper.

## Supporting information

S1 Fig Fertility and population under alternative scenarios for long-run total fertility rate and speed of convergence. The figure shows the global total fertility rate (TFR) (left) and projected population size (right) over time from 2025 until 3000. Each line represents a separate scenario of long-run TFR. Each row represents a different “convergence speed”, i.e. the rate at which each country’s TFR increases or decreases each period between 2100 (the end of WPP projections) and the date at which TFR reaches the scenario’s long-run TFR. The very slow convergence speed is 1%, the medium convergence speed is 3%, and the very fast convergence speed is 5%. In all scenarios, life expectancy at birth increases to 100 years.
(TIF)

S2 Fig Age-specific fertility rates globally and in selected countries. This figure demonstrates that countries converge to distinct age-specific fertility rates (ASFRs) despite that they all reach the same long-run total fertility rate.
(TIF)

S3 Fig The main rebound results are robust to different fertility assumptions. The figure displays projections of global total fertility rate (TFR) (left) and population size (right) over time from 2025 until 2400. For each line, global TFR converges to a specified level after 2100 until “rebounding” to replacement level TFR (about 2.05 children per woman) beginning at 2200. In all scenarios, after 2100, TFR increases or decreases at the “medium” convergence speed of 3% per five-year period until reaching the long-run level or 2200, whichever is earlier. All scenarios also assume that life expectancy at birth increases to 100 years.
(TIF)

S4 Fig Person-years lived (_5_*L*_*x*_) and life expectancy at birth (*e*_0_) under alternative mortality assumptions. Panel (a) displays the global average number of years a person would live in each age group if exposed to the mortality rates of the age group, as life expectancy rises to 100 years. Panel (b) displays life expectancy at birth under two distinct mortality assumptions. The assumption employed in the main results is that life expectancy at birth increases to 100 years. The panel also displays an alternative assumption, in which life expectancy increases to 120 years.
(TIF)

S5 Fig The main depopulation results are robust to alternative mortality assumptions. The figure demonstrates the robustness of the depopulation scenarios in Fig 1 Panel (a) of the main paper, given a 20% higher life expectancy (120 versus 100). Population size is highly sensitive to fertility assumptions and relatively insensitive to mortality assumptions. Very large increases in human lifespans would have only a small impact on long-term depopulation relative to differences in possible future fertility rates.
(TIF)

S6 Fig The main rebound results are robust to different mortality assumptions. This figure mimics Fig 5 from the main text, except that it presents global population size when long-run life expectancy is 100 years (left axis) or 120 years (right axis).
(TIF)

S1 Data The main rebound results are robust to different mortality assumptions. This figure mimics Fig 5 from the main text, except that it presents global population size when long-run life expectancy is 100 years (left axis) or 120 years (right axis).
(TXT)

## References

[1] Basten S, Lutz W, Scherbov S. Very long range global population scenarios to 2300 and the implications of sustained low fertility. Demographic Research. 2013;28:1145–1166. doi: 10.4054/DemRes.2013.28.39

[2] Gerland P, Raftery AE, Ševčíková H, Li N, Gu D, Spoorenberg T, et al. World population stabilization unlikely this century. Science. 2014;346(6206):234–237. doi: 10.1126/science.1257469 25301627 PMC4230924

[3] UN DESA. World Population Prospects 2022; 2022.

[4] Adsera A. Changing fertility rates in developed countries. The impact of labor market institutions. Journal of population economics. 2004;17:17–43. doi: 10.1007/s00148-003-0166-x

[5] Sobotka T, Beaujouan É, Van Bavel J. Introduction: Education and fertility in low-fertility settings. Vienna Yearbook of Population Research. 2017;15:1–16.

[6] Park N, Vyas S, Broussard K, Spears D. Near-universal marriage, early childbearing, and low fertility: India’s alternative fertility transition. Demographic Research. 2023;48:945–956. doi: 10.4054/DemRes.2023.48.34 38288421 PMC10824390

[7] Gietel-Basten S, Spears D, Visaria L. Low fertility with low female labor force participation in South India. Working Paper presented at International Population Conference, Hyderabad, India, December 5‒10, 2021. 2021;.

[8] Bryant J. Theories of fertility decline and the evidence from development indicators. Population and development review. 2007; p. 101–127. doi: 10.1111/j.1728-4457.2007.00160.x

[9] Lutz W, Skirbekk V, Testa MR. The low-fertility trap hypothesis: Forces that may lead to further postponement and fewer births in Europe. Vienna Yearbook of Population Research. 2006; p. 167–192.

[10] Raftery AE, Ševčíková H. Probabilistic population forecasting: Short to very long-term. International Journal of Forecasting. 2023;39(1):73–97. doi: 10.1016/j.ijforecast.2021.09.001 36568848 PMC9783793

[11] United Nations, Department of Economic and Social Affairs, Population Division. The United Nations on World Population in 2300. Population and Development Review. 2004;30(1):181–187. doi: 10.1111/j.1728-4457.2004.00009.x

[12] Kaneda T, Haub C. How many people have ever lived on Earth? Population Reference Bureau. 2022;.

[13] Marois G, Gietel-Basten S, Lutz W. China’s low fertility may not hinder future prosperity. Proceedings of the National Academy of Sciences. 2021;118(40):e2108900118. doi: 10.1073/pnas.2108900118 PMC8501780 34580226

[14] Galor O. The Journey of Humanity: The Origins of Wealth and Inequality. Penguin; 2022.

[15] Vollset SE, Goren E, Yuan CW, Cao J, Smith AE, Hsiao T, et al. Fertility, mortality, migration, and population scenarios for 195 countries and territories from 2017 to 2100: a forecasting analysis for the Global Burden of Disease Study. The Lancet. 2020;396(10258):1285–1306. doi: 10.1016/S0140-6736(20)30677-2 32679112 PMC7561721

[16] Gietel-Basten S, Sobotka T, Zeman K, et al. Future fertility in low fertility countries. In: World Population and Human Capital in the Twenty-First Century: An Overview. Oxford; 2014.

[17] Arenberg S, Kuruc K, Franz N, Vyas S, Lawson N, LoPalo M, et al. Research Note: Intergenerational Transmission Is Not Sufficient for Positive Long-Term Population Growth. Demography. 2022;59(6):2003–2012. doi: 10.1215/00703370-10290429 36259676 PMC10355194

[18] Vogl TS. Intergenerational associations and the fertility transition. Journal of the European Economic Association. 2020;18(6):2972–3005. doi: 10.1093/jeea/jvaa006

[19] Kolk M, Cownden D, Enquist M. Correlations in fertility across generations: can low fertility persist? Proceedings of the Royal Society B: Biological Sciences. 2014;281(1779):20132561. doi: 10.1098/rspb.2013.2561 24478294 PMC3924067

[20] Gietel-Basten S. The “Population Problem” in Pacific Asia. Oxford University Press; 2019.

[21] Hartmann B. Reproductive Rights and Wrongs: The Global Politics of Population Control. South End Press; 1995.

[22] Connelly M. Fatal misconception: The struggle to control world population. Harvard University Press; 2010.

[23] Gietel-Basten S, Rotkirch A, Sobotka T. Changing the perspective on low birth rates: why simplistic solutions won’t work. BMJ. 2022;379. doi: 10.1136/bmj-2022-072670 36379520 PMC10915774

[24] Jones CI, Romer PM. The new Kaldor facts: ideas, institutions, population, and human capital. American Economic Journal: Macroeconomics. 2010;2(1):224–245.

[25] Preston SH, Heuveline P, Guillot M. Demography: Measuring and Modeling Population Processes. Blackwell Publishers; 2001.

