# Supplementary figures and images for: Long-term population projections: Scenarios of low or rebounding fertility
Source: PLoS One. 2024 Apr 4;19(4):e0298190. doi: 10.1371/journal.pone.0298190 (PMC10994275; doi:10.1371/journal.pone.0298190)

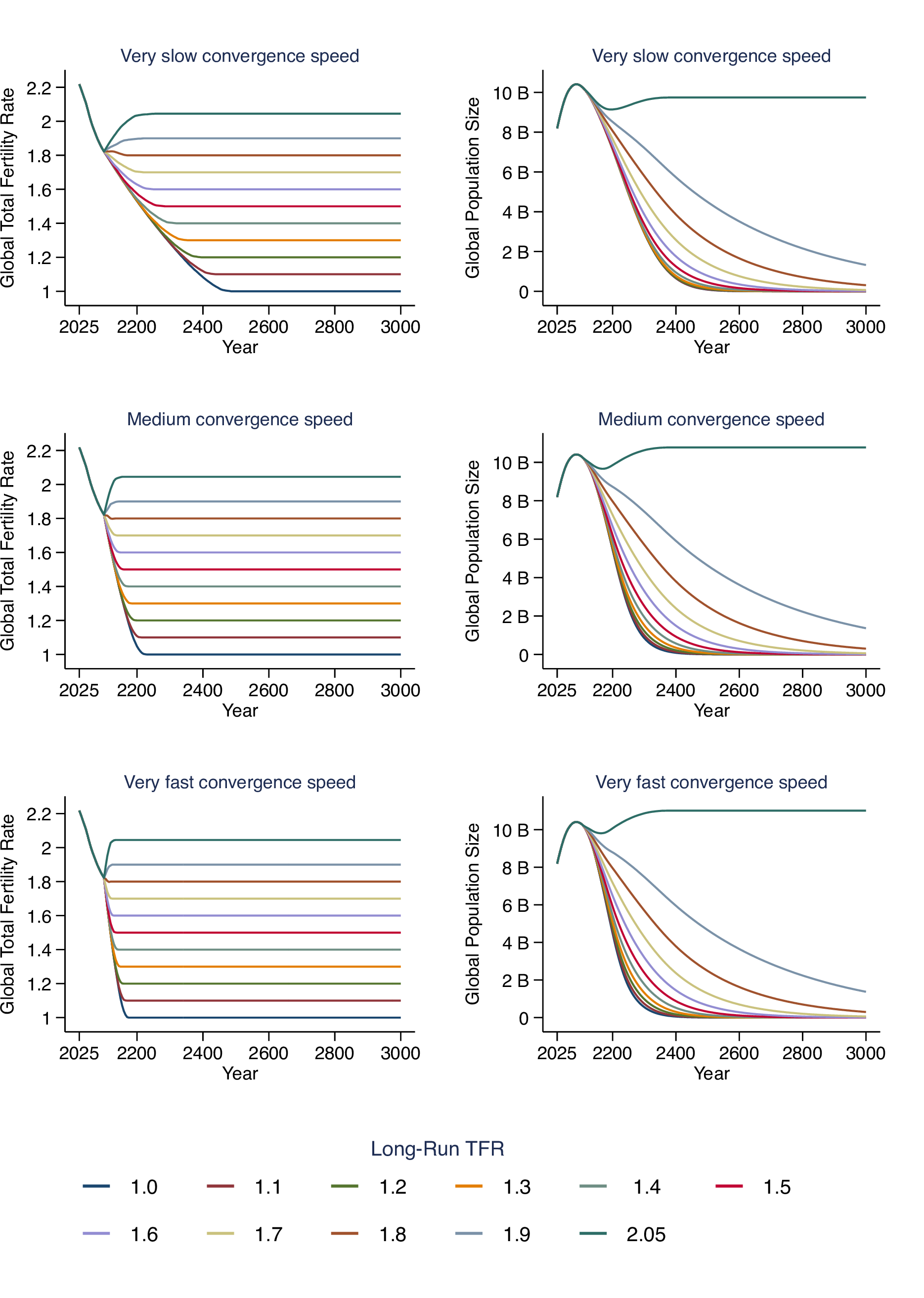

Supplement: S1 Fig — The figure shows the global total fertility rate (TFR) (left) and projected population size (right) over time from 2025 until 3000. Each line represents a separate scenario of long-run TFR. Each row represents a different “convergence speed”, i.e. the rate at which each country’s TFR increases or decreases each period between 2100 (the end of WPP projections) and the date at which TFR reaches the scenario’s long-run TFR. The very slow convergence speed is 1%, the medium convergence speed is 3%, and the very fast convergence speed is 5%. In all scenarios, life expectancy at birth increases to 100 years. (TIF) [file pone.0298190.s001.tif]

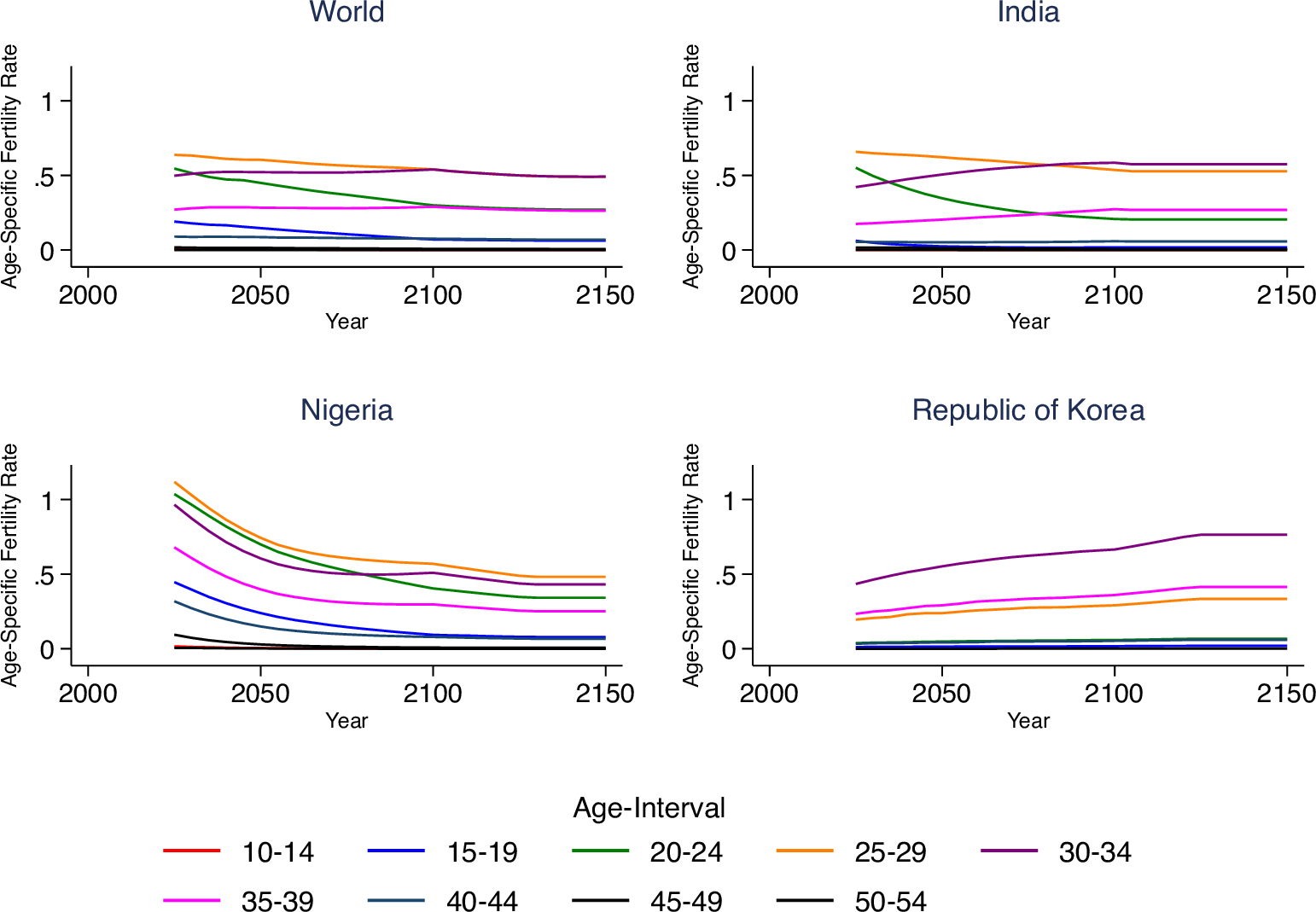

Supplement: S2 Fig — This figure demonstrates that countries converge to distinct age-specific fertility rates (ASFRs) despite that they all reach the same long-run total fertility rate. (TIF) [file pone.0298190.s002.tif]

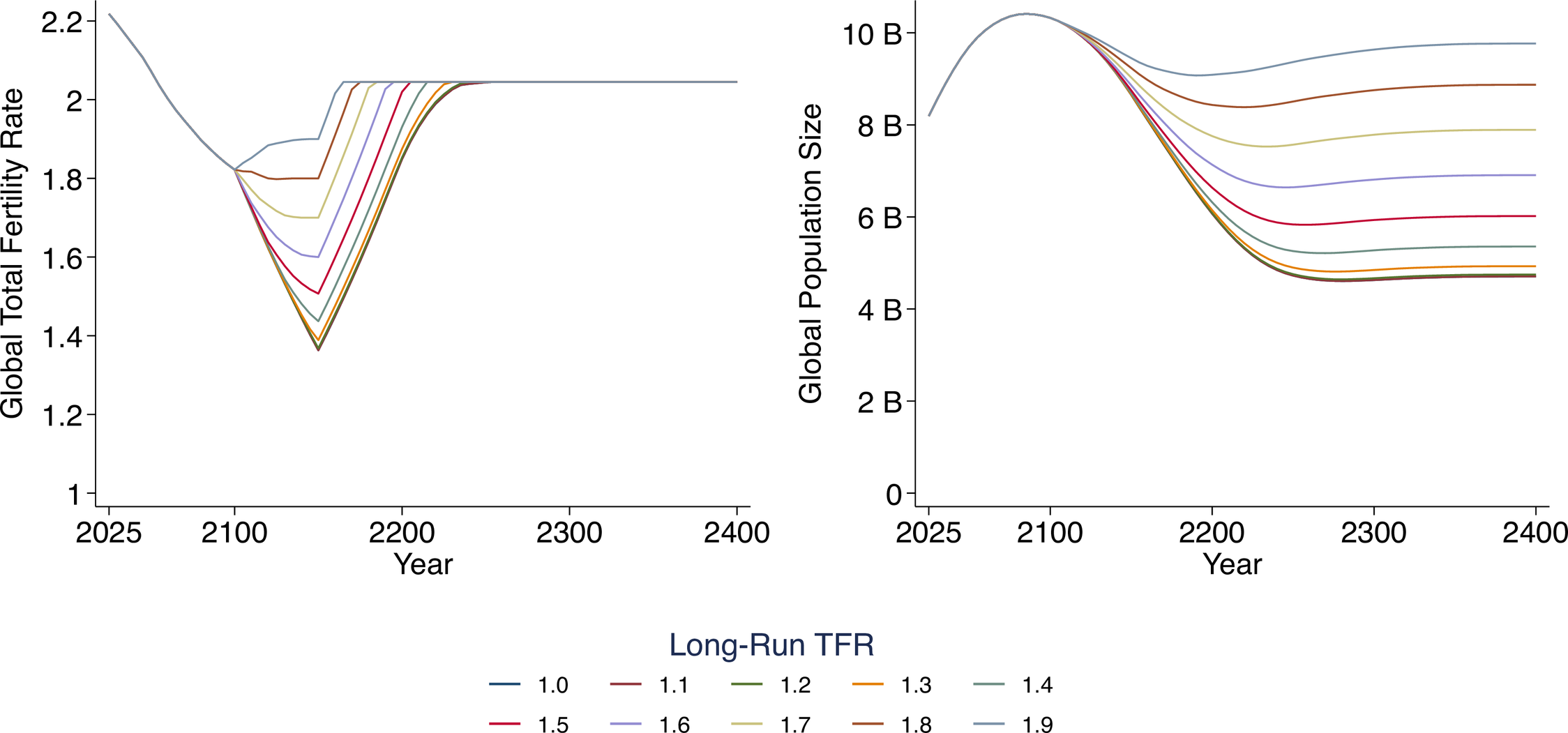

Supplement: S3 Fig — The figure displays projections of global total fertility rate (TFR) (left) and population size (right) over time from 2025 until 2400. For each line, global TFR converges to a specified level after 2100 until “rebounding” to replacement level TFR (about 2.05 children per woman) beginning at 2200. In all scenarios, after 2100, TFR increases or decreases at the “medium” convergence speed of 3% per five-year period until reaching the long-run level or 2200, whichever is earlier. All scenarios also assume that life expectancy at birth increases to 100 years. (TIF) [file pone.0298190.s003.tif]

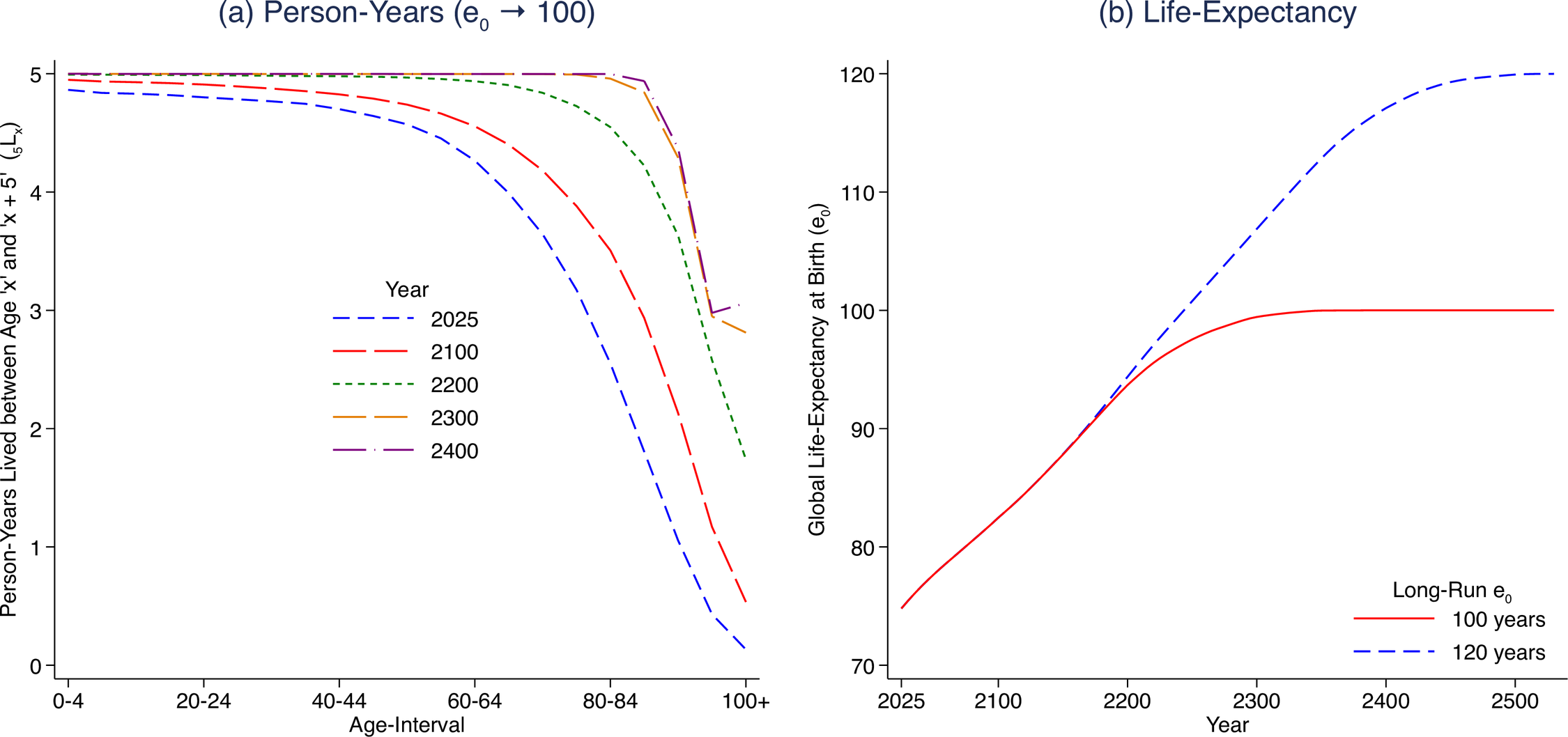

Supplement: S4 Fig — Panel (a) displays the global average number of years a person would live in each age group if exposed to the mortality rates of the age group, as life expectancy rises to 100 years. Panel (b) displays life expectancy at birth under two distinct mortality assumptions. The assumption employed in the main results is that life expectancy at birth increases to 100 years. The panel also displays an alternative assumption, in which life expectancy increases to 120 years. (TIF) [file pone.0298190.s004.tif]

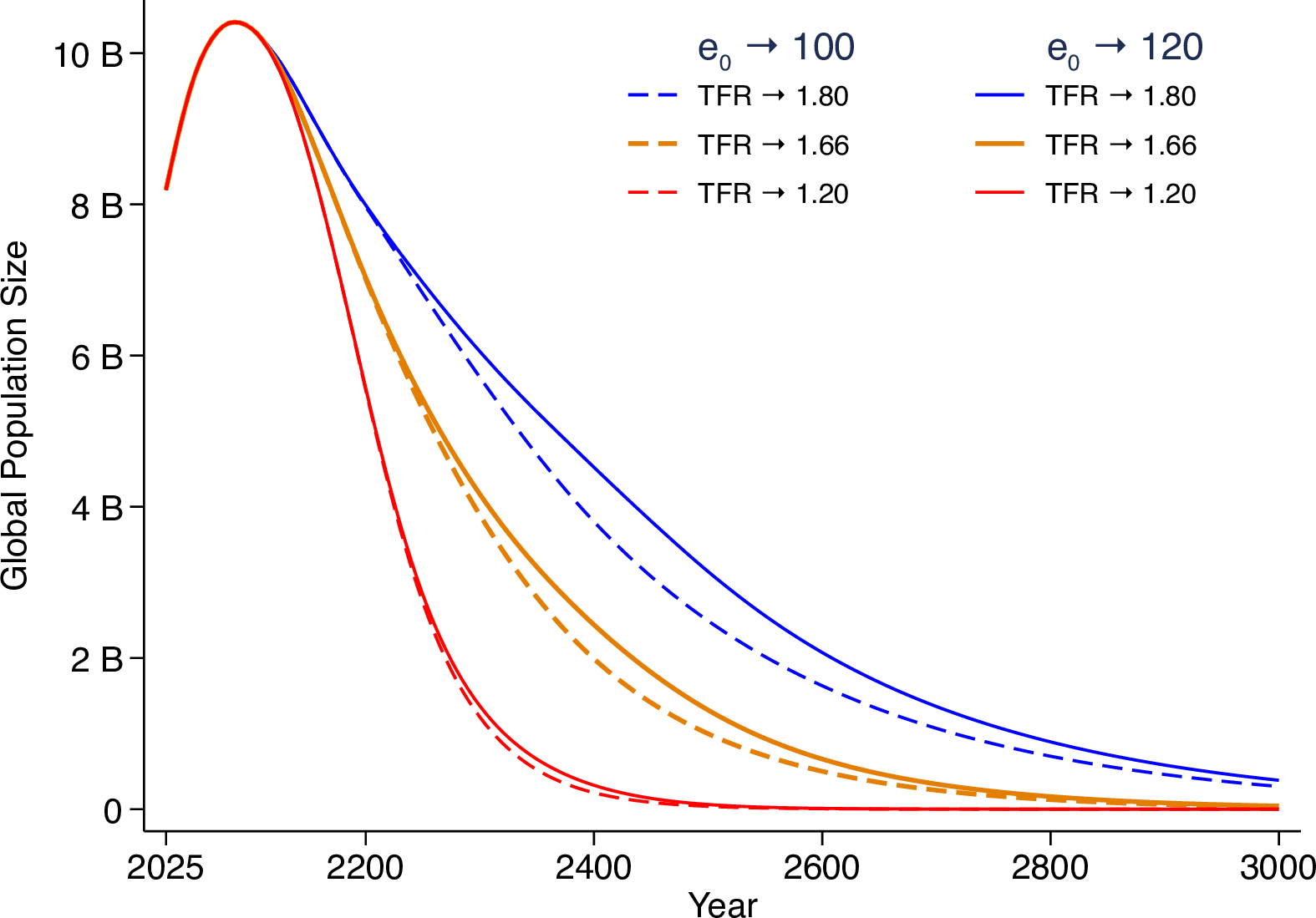

Supplement: S5 Fig — The figure demonstrates the robustness of the depopulation scenarios in Fig 1 Panel (a) of the main paper, given a 20% higher life expectancy (120 versus 100). Population size is highly sensitive to fertility assumptions and relatively insensitive to mortality assumptions. Very large increases in human lifespans would have only a small impact on long-term depopulation relative to differences in possible future fertility rates. (TIF) [file pone.0298190.s005.tif]

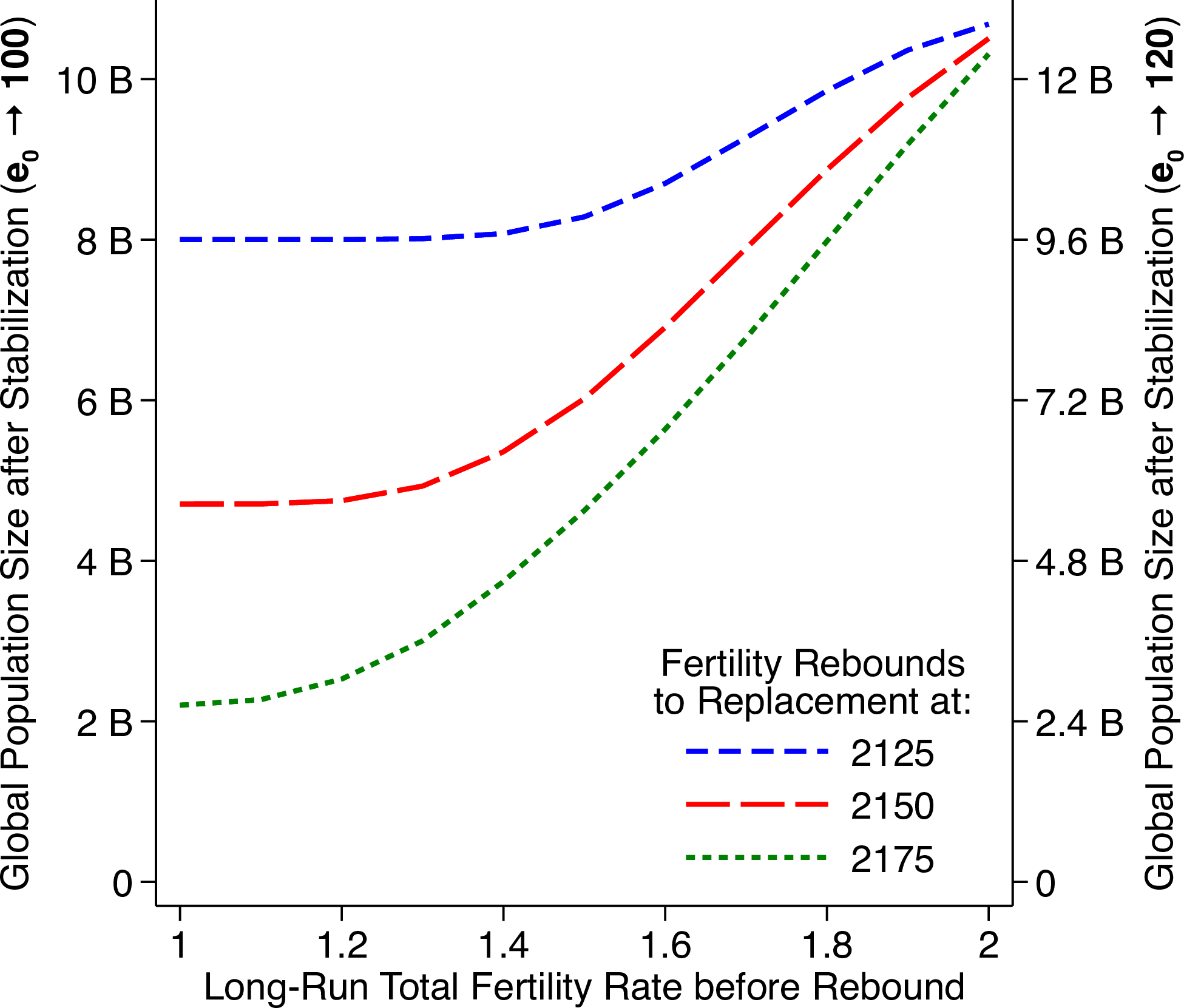

Supplement: S6 Fig — This figure mimics Fig 5 from the main text, except that it presents global population size when long-run life expectancy is 100 years (left axis) or 120 years (right axis). (TIF) [file pone.0298190.s006.tif]
